# Multi-Plant Screening and Controlled Evaluation of Two Selected Plant Species Against *Tuta absoluta* (Meyrick)

**DOI:** 10.3390/insects17090977

**Published:** 2026-09-21

**Authors:** Abubakar Mshora, Marco Mng’ong’o, Luisa Santos, Tomás Chiconela

**Affiliations:** 1 Department of Crop Science and Horticulture, College of Agricultural Science and Technology, Mbeya University of Science and Technology (MUST), Mbeya P.O. Box 131, Tanzania; marcojohn10@gmail.com; 2 Department of Plant Protection, Faculty of Agronomy and Forestry Engineering, Eduardo Mondlane University (UEM), Maputo P.O. Box 257, Mozambique; luisasantos47@gmail.com (L.S.); tfchiconela@gmail.com (T.C.)

**Keywords:** ecological pest management, tomato, biological control, push–pull strategy, *Tuta absoluta*

## Abstract

*Tuta absoluta* is an invasive species that has managed to expand beyond its native geographical area and causes severe yield losses in tomato. Despite the application of synthetic insecticides in getting rid of the pest, farmers have reported reduced efficacy over the course of time due to resistance. With regard to ecological pest management approaches, studies regarding the use of companion plants in the management of *T. absoluta* are scarce. Therefore, we screened fourteen potential companion plants for repellency using a Y-tube olfactometer followed by cage and screenhouse experiments. Data were analyzed using R software (version 4.6.0; R Core Team, 2026). The screening results showed that *Allium schoenoprasum* and *Aeollanthus fruticosus* significantly repelled *T. absoluta* compared to other potential companion plants at *p* < 0.001. In a dual-choice cage experiment, tomato plants placed with *A. schoenopraum* and *A. fruticosus* were recorded to have lower leaf damage percentages of 47.27% and 33.94%, respectively, compared to the negative control (66.34%). Similarly, tomato plants intercropped with *A. schoenopraum* and *A. fruticosus* showed lower leaf damage percentages of 13.92% and 25.00%, respectively, compared to the negative control (63.38%) under the screenhouse experiment. Therefore, *A. schoenopraum* and *A. fruticosus* exhibit significant potential in the management of *T. absoluta* in tomato.

## Simple Summary

*Tuta absoluta* (Meyrick) is one of the most destructive insect pests which infests solanaceous plants, posing a substantial threat to tomatoes (*Solanum lycopersicum*) through the devastation of their yields. The larvae of *T. absoluta* attack during all the life stages of tomato plants, leading to leaf discoloration and holes in fruits. Currently, most farmers manage *T. absoluta* using tank-mixed synthetic insecticides, which subsequently pose risks to the environment and biodiversity. Integrated pest management approaches using biological, cultural, physical and low-toxicity chemical methods have been recently promoted. However, companion plants used in the management of *T. absoluta* are scarce and documentation is limited. Therefore, this study screened fourteen potential companion plants in the lab followed by trial confirmation in insect-proof mesh cages and a pest exclusion screenhouse. The results indicated that *Allium schoenoprasum* and *Aeollanthus fruticosus* successfully managed *T. absoluta* by repelling it, leading to low oviposition and leaf damage, both in cage and screenhouse experiments. These results indicate the potential of *A. schoenoprasum* and *A. fruticosus* for the integrated, ecological management of *T. absoluta.* However, an empirical investigation under an open-field environment in diverse agroecological zones is warranted. Likewise, further studies are essential to check their compatibility with natural enemies and entomopathogens.

## 1. Introduction

Tomato Leaf Miner (*Tuta absoluta* (Meyrick) Lepidoptera: Gelechiidae) is a highly destructive insect pest that significantly threatens food security and livelihoods across various continents including Europe, Asia and Africa [1]. *T. absoluta* originated in Peru and managed to spread to various continents like Europe, Asia, the Mediterranean coastline, and, most recently, Africa [2]. The pest has been documented to infest several solanaceous plants like potato (*Solanum tuberosum* L.), pepper (*Capsicum annum* L.) and eggplant (*Solanum melongena* L.), with the highest damage recorded from the vegetative to reproductive growth stages of tomato (*Solanum lycopersicum* L.) [3,4].

Currently, most farmers use synthetic insecticides repetitively to control *T. absoluta* as their first option [5,6]. However, synthetic insecticides have been reported to be ineffective in the management of *T. absoluta* due to the feeding nature of larvae [7]. This has forced farmers to over-spray and has subsequently led to the development of insecticide resistance and the accumulation of insecticide residues in the environment, which in turn affects biodiversity [8,9]. This drives a need for the development of eco-friendly and integrative approaches to tackle the above-mentioned problems [10].

The most effective and commonly known environmentally friendly approach is biological control through the use of natural enemies like predators and parasitoids, sex pheromones as well as bio-pesticides [11,12]. For example, Biondi et al. [1] and Silva et al. [13] reported about the high effectiveness of Mirid bugs such as *Macrolophus basicornis* Stål, *Engytatus varians* Distant and *Dicyphus* spp. Fieber in preying on the eggs and young larvae of *T. absoluta*. Likewise, several native populations of parasitoid *Bracon nigricans* Szépligeti have been documented to effectively control *T. absoluta* in Tunisia and Kenya [11,14].

Also, Parolin et al. [15] reported about the good performance of pheromone-based mass trapping and mating disruption in managing *T. absoluta*. However, the integrated pest management strategy using eco-friendly methods with minimal application of synthetic insecticides ensures the sustainable management of *T. absoluta* [12]. For example, Mansour et al. [11] reported that the combination of natural enemies and entomopathogens significantly controlled *T. absoluta* compared to when the two approaches were used as stand-alone treatment.

Several success stories have been documented regarding the effectiveness of companion plants integrated with primary crops in the management of various insect pests [15,16]. For example, Sun et al. [17] reported about the potential of *Rosmarinus officinalis* L. in the management of *T. absoluta* by reducing egg-laying and hatching rates in microscopic, cage and greenhouse experiments. Likewise, Li et al. [18] reported about the reduction in egg laying and settling of female moth *Frankliniella occidentalis* Pergande on the target host integrated with *Rosmarinus officinalis*.

Also, Beck et al. [19] reported that the companion plants work by releasing volatile organic compounds (semio-chemicals), which confuse insect pests and subsequently protect the primary crop. On the other side, a laboratory study conducted by Dardour et al. [20] revealed that the volatile organic compounds released by marigold (*Tagetes patula* L.) and basil (*Ocimum basilicum* L.) significantly repelled green peach aphid (*Myzus persicae* Sulzer) on green pepper (*Capsicum annum* L.). Likewise, Conboy et al. [21] revealed the significant reduction in greenhouse whitefly (*Trialeurodes vaporariorum* Westwood) population when tomato crop was intercropped with marigold (*Tagetes minuta*) due to the high concentration of monoterpene limonene.

Moreover, companion plants can be used as attractants through trapping natural enemies, which subsequently reduce damage to the main crop under a specific planting pattern [22]. For instance, Duraimurug et al. [23] revealed the attraction behaviours of okra (*Abelmoschus esculentus* L.) and maize (*Zea mays* L.) to *Helicoverpa armigera* Hübner adults, which significantly protect the primary crops from being infested.

Furthermore, *Plectranthus amboinicus* L. significantly repelled *Bemisia tabaci* and minimized leaf damage on the tomato crop [24]. Likewise, Tang et al. [25] and Wang et al. [26] reported about the attraction properties of companion plants to natural enemies, which significantly reduced damage in host plants compared to mono-cropping.

The increasing awareness of the application of eco-friendly methods has resulted in exploration into how effective companion plants manage insect pests. For example, the previous investigations documented the potentials of *R. officinalis*, *Tagetes erecta*, and *Allium cepa* L. in the management of *T. absoluta*, *Spodoptera litura* Fabricius and *Plutella xylostella* L., respectively [17,27,28]. Plant diversification is among the most promising methods in pest management through repelling insect pests and/or attracting natural enemies [26,29]. However, there are limited studies regarding the use of companion plants in the management of *T. absoluta* in tomato.

The primary objective of this study was to evaluate potential companion plants for the ecological management of *T. absoluta* in tomato. To achieve this, we first screened 14 potential companion plants in the lab using a Y-tube olfactometer. Two screened companion plants (*Allium schoenoprasum* L. and *Aeollanthus fruticosus* Gürke) were evaluated under exclusion cage experiments followed by protective screenhouse experiments.

## 2. Materials and Methods

### 2.1. Study Area

**Figure 1:**
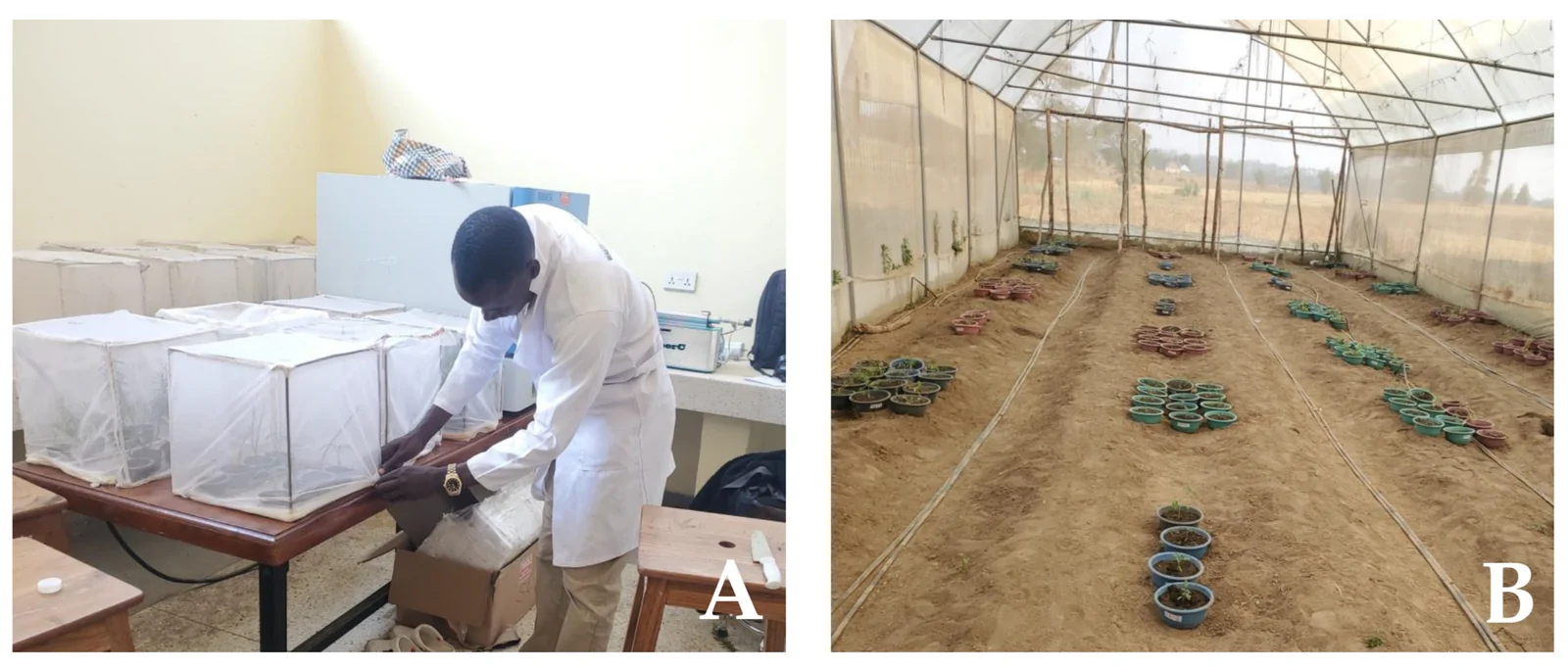
A picture showing (**A**) MUST laboratory experiments and (**B**) MUST screenhouse experiments.

This study was conducted in Mbeya Region (8°54′33.84″ S, 33°27′38.88″ E), Tanzania (Figure 1). The screening and cage trials were conducted at Mbeya University of Science and Technology (MUST) laboratories, and screenhouse experiment was conducted at MUST demonstration farm located in Mbeya Urban district.

### 2.2. Collection and Rearing of Tuta absoluta

The population of *T. absoluta* was collected from infested tomato field (l8°95′13.7″ S, 33°42′64.9″ E) in Mbeya Urban district. A minimum of 50 leaves attacked by *T. absoluta* were randomly sampled using the standardized protocol of two leaves per plant, whereby one leaf was taken from the upper canopy and another one from the lower canopy of the tomato plant. This sample size was chosen to optimize statistical power while reducing the intra-plant spatial autocorrelation and sampling bias. Samples were put in polyethene plastic bags and then sent to MUST laboratory. To ensure only *T. absoluta* was taken, leaves were carefully checked using a binocular stereo microscope (VisiScope^®^ SBT150; VWR International, Radnor, PA, USA).

For colony establishment, larvae of *T. absoluta* on infested tomato plants were incubated in ventilated plastic container. The pupae were housed in 45 cm × 45 cm × 45 cm fine nylon mesh cage (160 μm mesh aperture, 150 mesh/inch^2^; BugDorm, MegaView Science, Taiwan, China) containing whole potted tomato plants for maintenance of the colony. The rearing cages were maintained within a bioclimatic growth chamber under a standardized environmental condition of 25 ± 1 °C, 65 ± 5% RH, and 12:12 h (L:D) photoperiods. The adult insects received a 10% aqueous sucrose solution to provide baseline energetic requirement for mating and oviposition.

### 2.3. Preparation of Potential Companion Plants

The potential companion plants were selected based on local availability, economic value and climatic adaptability in the Southern Highland region of Tanzania. About 14 common reported potential companion plants obtained from an extensive literature review and farmer survey study (data were not included in this study) were subjected to screening using a Y-tube olfactometer assay choice.

All selected potential companion plants that are reported to possess insecticidal properties against various insect pests are presented as follows: Spear Mint (*Mentha spicata*) [30,31], Oregano (*Origanum vulgare*) [32,33,34], Rosemary (*Rosmarinus officinalis*) [17,35], Chives (*Allium schoenoprasum*) [36,37], leek (*Allium ampeloprasum* L.) [38,39], Lemon grass (*Cymbopogon citratus* L.) [40,41,42], Mexican marigold (*Tagetes erecta*) [27,43,44], Pyrethrum (*Tanacetum cinerariifolium* L.) [45,46], African catmint (*Aeollanthus fruticosus*) [47], Rape (*Brassica napus* L.) [48], Common onion (*Allium sativum* L.) [49,50], Garlic (*Allium cepa*) [28,50,51], Peppermint (*Mentha piperita* L.) [52,53,54], Lemon thyme (*Thymus citriodorus* L.) [55], and Cardamom (*Ellettaria cardamomum* L.) [56,57,58] against tomato (DHAHABU F1 variety).

*R. officinalis* was included in this trial as a positive control because it was confirmed by Sun et al. [17] to be effective against *T. absoluta* in tomato crop under a Y-tube olfactometer assay choice and cage experiments.

The potential companion plant seedlings were purchased from the JJ seedling company, Morogoro, Tanzania, and used in experiment at 6 weeks old. The potential companion plants were used strictly to vegetative growth stage. Flowering individuals were excluded to prevent variations in olfactory cues that could influence insect behaviour. Tomato seedlings were initiated from seeds at MUST demonstration farm and used at 21 days old from sowing.

### 2.4. Behavioral Responses of T. absoluta to Selected Potential Companion Plants

The responses of adult *T. absoluta* to selected potential companion plant volatiles were examined in a Y-tube olfactometer (arm = 10 cm, stem = 14 cm, diameter = 3 cm). Charcoal-purified and humidified air was passed into the two arms of the olfactometer, each at a flow rate of 350 mL/min. An electric-powered air-free vacuum pump (Model: DAA-V174-EB, GAST manufacturing company, Benton Harbor, MI, United States) with modification was used to suck air out of the Y-tube at a flow rate of 700 mL/min.

Because *T. absoluta* is nocturnal, a night condition was simulated by placing a red fluorescent bulb about 2 m above the olfactometer, which emits about 1000 Lux illuminations of red light. The response of *T. absoluta* adults to volatiles from host and non-host plants was tested in assays. The host and no-host-plant headspace volatiles were offered into the two arms of the Y-tube olfactometer from the roasting bags containing the plants. To ensure that only headspace plant volatiles were released into the roasting bags, the whole pot containing the soil and the plant was covered with aluminum foil to the base of the plant.

Sixty adults within three days of emergence (male-to-female ratio, 1:1) were evaluated across six true biological replicates (*n* = 10 insects per biological replicate). To eliminate lingering chemical cues and avoid confounding environmental biases, a fresh plant was introduced at the beginning of each biological replicate (after every 10 insects). Individual insects were tested completely independently and used only once. Each insect was given 10 min to make a choice. An insect was considered to have made a choice when it walked at least 5 cm into one of the arms and stayed for two or more minutes. The Y-tube was cleaned after every 5 insects tested, and the arms were then switched. The individual choice behaviour of an insect was defined as primary observational unit, while biological replicate was integrated into the analysis to account for potential batch effects and prevent pseudo-replication. All the bioassays were conducted under controlled laboratory conditions (27 ± 1 °C and 70 ± 5% RH).

### 2.5. Effect of Allium schoenoprasum and Aeollanthus fruticosus on T. absoluta in Cage Experiments

The dual-choice and multiple-choice tests were conducted following the method described in [59], with some modifications. In each cage experiment, 6-week-old seedlings were used, in which the dual-choice test was conducted using two potted plants (one potted tomato seedling and one potted companion plant), and the multiple-choice test was carried out using three potted plants (two potted tomato seedlings and one potted companion plant).

**Figure 2:**
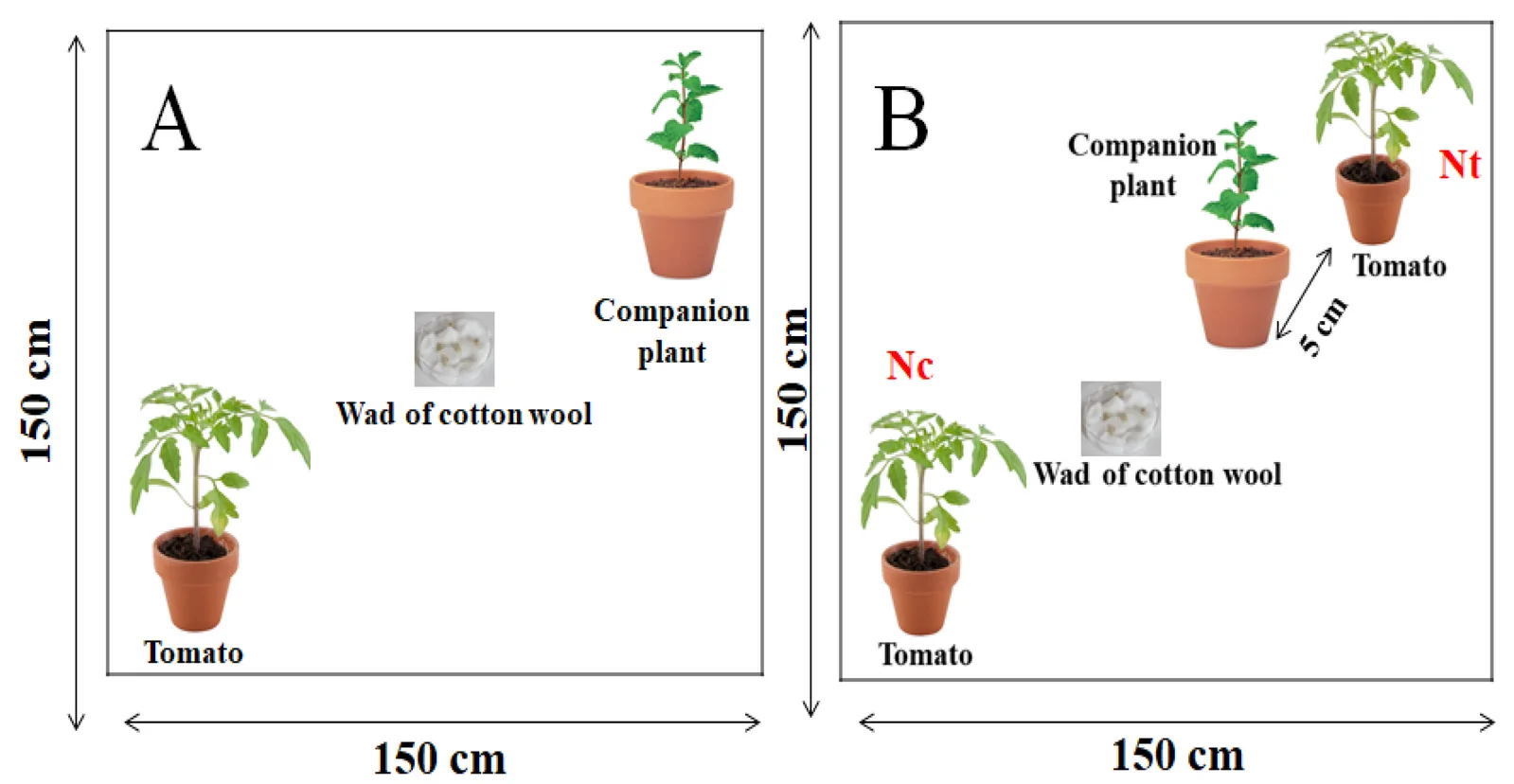
Cage experimental layout for (**A**) dual-choice test and (**B**) multiple-choice test. Nc means control tomato group and Nt means treated tomato group.

Both dual-choice and multiple-choice tests involved three plant combinations placed in opposite corners of an oviposition cage (150 cm × 150 cm × 150 cm), covered with fine-wire mesh netting. For the host plant (tomato) + companion plant treatment, the companion plant was behind the host plant with a distance of 5 cm (Figure 2).

Ten gravid *T. absoluta* females (2–3 days) were released into the cage to oviposit overnight, and a cotton ball saturated with 10% honey solution in a Petri dish (90 mm × 15 mm) was placed in the middle of the cage to serve as food source for the adult moths. On the following day, the eggs and egg batches on each plant were collected and counted using a stereo microscope (Leica EZ4HD, Leica Microsystems, and Schweiz, Switzerland) for about 3 days. The percentages of leaf damage caused by *T. absoluta* larvae were recorded after 4 days up to the 10th day.

Two screened plants (*A. fruticosus* and *A. schoenoprasum*) were selected based on the screening results, and *R. officinalis* was included as a positive control. Treatments used in the cage trials were T1: tomato alone (negative control); T2: tomato + *A. fruticosus*, T3: tomato + *A. schoenoprasum*, T4: tomato + *Rosmarinus officinalis* (positive control). The experiment consisted of ten biological replicates.

### 2.6. Calculations Involved Multiple-Choice Cage Experiment for Oviposition

$$\mathrm{Percent}\;\mathrm{effective}\;\mathrm{repellency}\;(\%\;\mathrm{ER})=\frac{Nc-Nt}{Nc}\times 100$$

$$\mathrm{Oviposition}\;\mathrm{Activity}\;\mathrm{Index}\;(\mathrm{OAI})=\frac{Nt-Nc}{Nt+Nc}$$

$$\mathrm{Oviposition}\;\mathrm{Deterrent}\;\mathrm{Index}\;(\mathrm{ODI})=\frac{Nc-Nt}{Nc+Nt}\times 100$$

where Nc = the mean number of eggs in the control group and Nt = the mean number of eggs in the test (treated) group.

### 2.7. Effect of A. schoenoprasum and A. fruticosus on T. absoluta in Screenhouse Experiments

**Figure 3:**
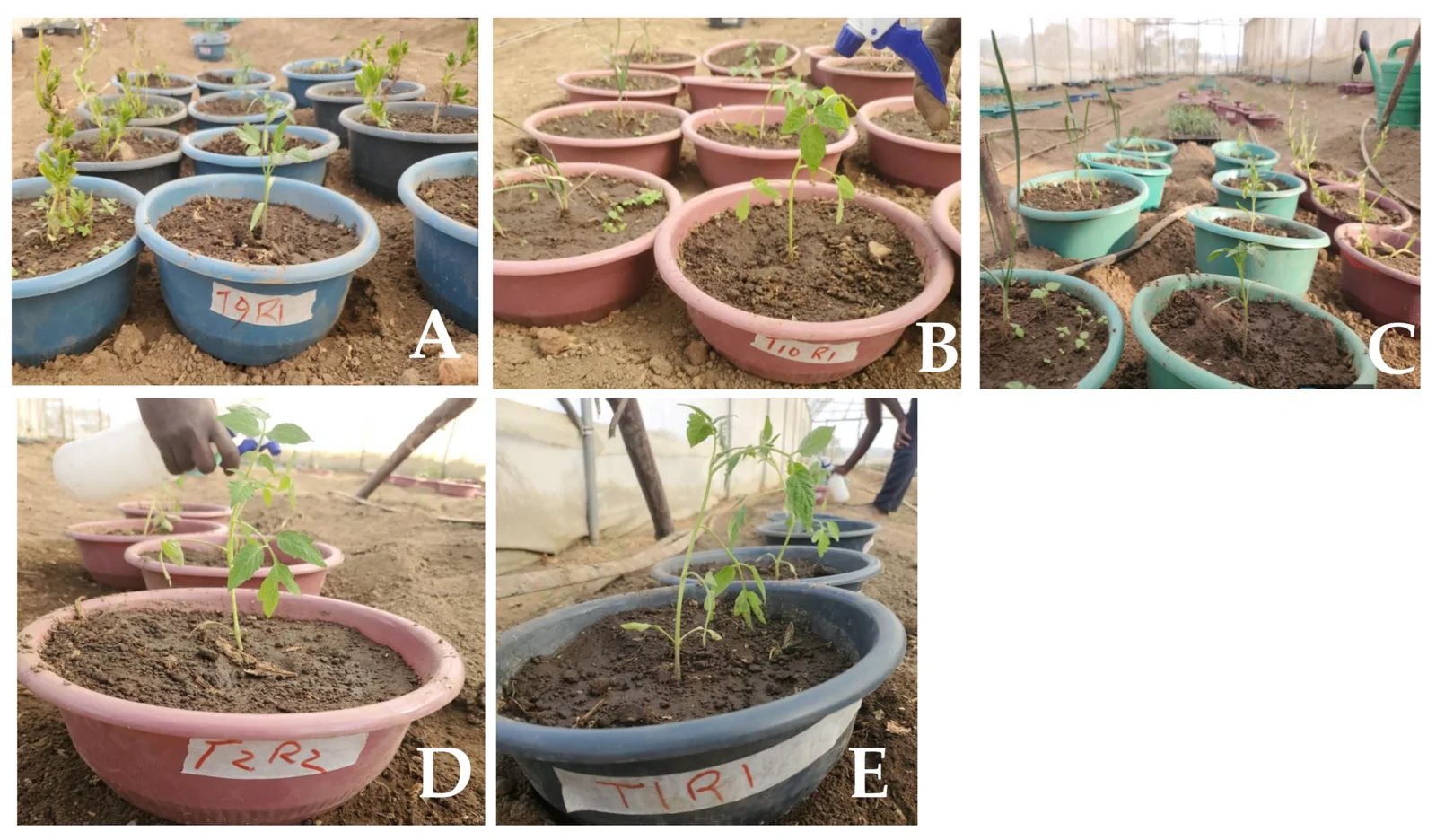
Screenhouse experimental layout for (**A**) tomato + *A. fruticosus*, (**B**) tomato + *A. schoenoprasum*, (**C**) tomato + *A. fruticosus* + *A. schoenoprasum*, (**D**) tomato + SNOW TIGER (positive control) and (**E**) tomato + water sprayer (negative control).

A randomized complete block design (RCBD) consisting of four distinct blocks to account for microclimatic gradients (light and ventilation) was used to assess the effects of different treatments on oviposition and leaf damage caused by *T. absoluta*. The tomato crop (DHAHABU F1) was used at 4 weeks old from sowing, while *A. schoenoprasum* and *A. fruticosus* plants were used at 6 weeks from cutting. The experiment was set to a 240 m^2^ screenhouse on 25th September, 2025. Similar to cage experiment, the two screened plants (*A. fruticosus* and *A. schoenoprasum*) were selected based on screening results. The experiment involved five treatments, which were T1: tomato + water sprayer (negative control); T2: tomato + SNOW TIGER (positive control); T3: tomato + *A. fruticosus;* T4: tomato + *A. schoenoprasum;* and T5: combined companion plants (tomato + *A. fruticosus* + *A. schoenoprasum*) with seven biological replicates (Figure 3).

The control treatments consisted of tomato alone sprayed with water (negative control) and tomato alone sprayed with synthetic pesticide SNOW TIGER (Chlorfenapyr 100 g/L) (positive control) at a concentration of 15 mL per 16 L of water using a knapsack sprayer once per week. In treatments consisting of companion plants, tomato plants were placed in the middle, surrounded by companion plants in 1:1:1 ratio [21] with modification. The distance between treatments within block was 1.25 m and between blocks was 1.5 m. Approximately 1000 gravid females of *T. absoluta* were introduced in the screenhouse from the laboratory culture. The number of eggs in each potted tomato was determined by visual inspection and with the aid of stereo microscope each day for about 3 days, and the number of leaves damaged by *T. absoluta* was recorded after 5 days. The number of eggs and damaged leaves on each tomato plant from the control group and intercropping group was counted individually.

### 2.8. Statistical Analysis

Data analyses were conducted using R software (version 4.6.0; R Core Team, 2026). Behavioral choice data from the Y-tube olfactometer bioassays were evaluated using Pearson chi-square Test of Independence (χ^2^) to analyze the overall association between treatment categories and insect directional responses (df = 14). Following the primary omnibus test, individual plant-to-control comparisons were isolated and evaluated using separate pairwise Chi-square goodness-of-fit tests against an expected null distribution of equal choice (50:50). To control for the inflation of Type I errors resulting from multiple simultaneous individual comparisons, all downstream *p*-values were adjusted using the Bonferroni correction method within R. Non-responding *T. absoluta* insects were not included in the analysis.

The oviposition and leaf damage percentages were fitted to Generalized Linear Mixed Models (GLMMs) by using glmmTMB package. Egg counts were analyzed using a negative binomial distribution (nbinom2 family, log link) to accommodate significant overdispersion in the raw counts. The response variable for leaf damage percentage was structured as a two-column binomial matrix consisting of the numerator (number of damaged leaves) and the corresponding denominator (total leaves evaluated per sampling unit) modeled using binomial distribution with a logit link function. The host treatments were treated as primary fixed effect in all models. In cage experiment, the cage ID was specified as a random effect, while the block ID and pot ID were specified as nested random intercepts in screenhouse experiment [60].

Model adequacy, overdispersion parameters, and residual homogeneity were verified using simulated residual diagnostics within the DHARMa package environment. Type III Wald chi-square tests were used to assess the fixed effect significance, and pairwise post hoc comparisons among treatments were conducted using Tukey’s honestly significant difference (HSD) test via the emmeans package. All the analyses were conducted at 5% probability level (*p* ≤ 0.05).

## 3. Results

### 3.1. Behavioral Responses of T. absoluta in Y-Tube Olfactometer Experiment

#### 3.1.1. Control Experiments

**Figure 4:**
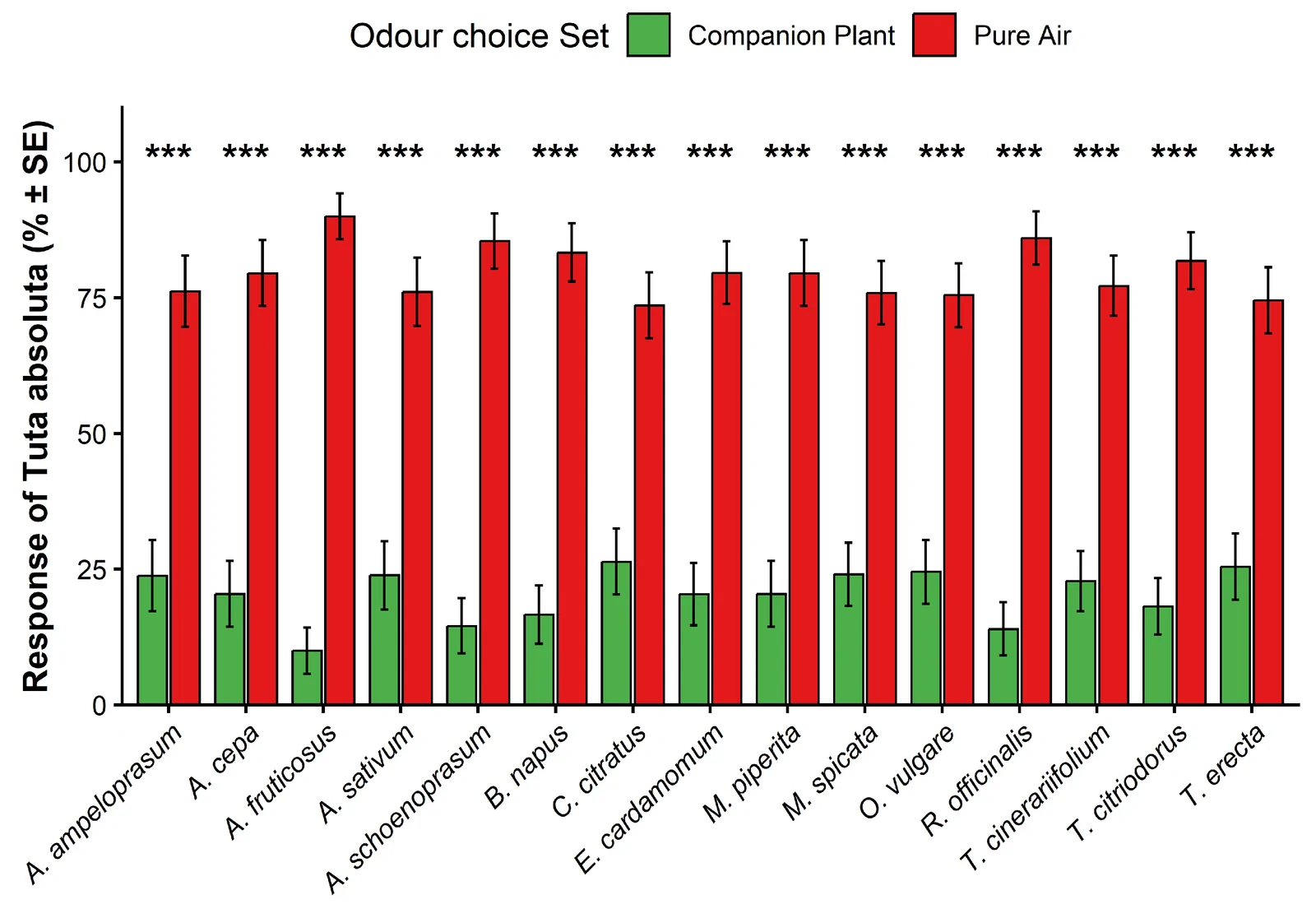
Responses (%) of adult *T. absoluta* to pure air and odors of companion plants. Asterisks indicate statistical significance levels: *** *p* < 0.001.

Control experiment 1 involved fourteen potential companion plants, with the air source (empty odor source) showing a significant difference at *p* ≤ 0.001, where most of the *T. absoluta* adults chose the empty odor source (Figure 4). Likewise, results from control experiment 2 involving two empty odor sources of Y-tube showed that the choice of *T. absoluta* adults in two arms did not significantly differ from one another, with responses of 47% and 53% at *p* > 0.05.

On the other side, control experiment 3 involving an empty odor source and tomato odor source differed significantly at *p* < 0.001, where the majority of *T. absoluta* adults (93.75%) preferred the tomato odor source, while a few (6.25%) chose the empty odor source. These results indicated that the modified Y-tube olfactometer instrument was effective for bioassay choice experiments involving potential companion plants versus tomato plants.

#### 3.1.2. Y-Tube Bioassay Choice Test for Treatments

**Figure 5:**
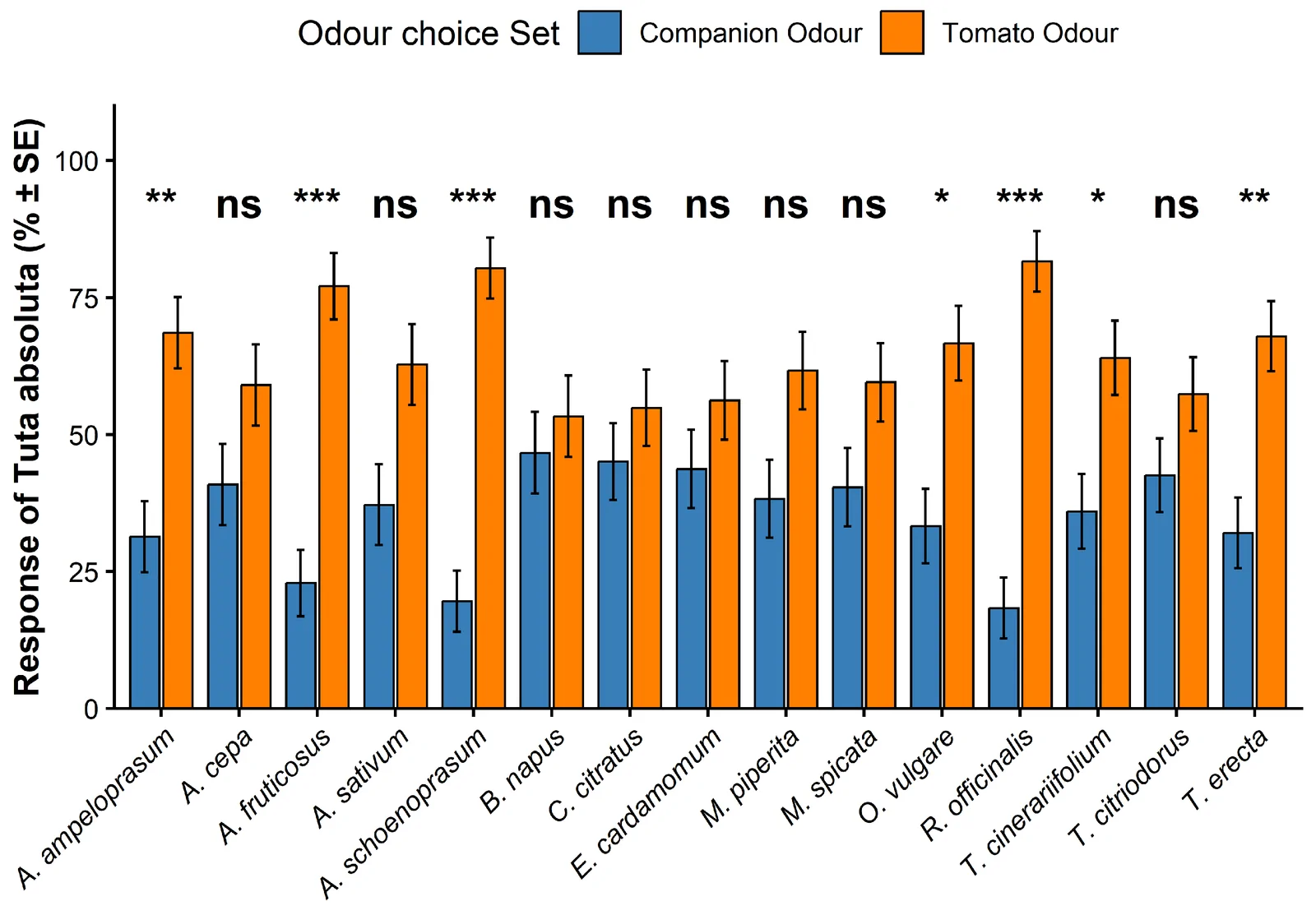
Responses (%) of adult *T. absoluta* to tomato and odors of companion plants. Asterisks indicate statistical significance levels: * *p* < 0.05, ** *p* < 0.01, *** *p* < 0.001 and ns indicates not significant.

The experiment involving screening of fourteen potential companion plants was tested using Pearson chi-square to examine the relationship between choice of insect and volatile organic compounds among the treatments. The results indicated a statistically significant association between choice of insect and odor of plant, χ^2^ (*df* = 14, N = 729) = 24.45, *p* = 0.04. The following potential companion plants significantly repelled *T. absoluta* adults: *Origanum vulgare* (*p* = 0.02), *Aeollanthus fruticosus* (*p* < 0.001), *Rosmarinus officinalis* (*p* < 0.001), *Allium schoenoprasum* (*p* < 0.001), *Allium ampeloprasum* (*p* = 0.008), and *Tagetes erecta* (*p* = 0.009). The rest of the potential companion plants did not significantly differ from the tomato odor source, which indicated low repellency effects (Figure 5).

### 3.2. Effects of Allium schoenoprasum and Aeollanthus fruticosus on Development of T. absoluta Under Cage Experiments

#### 3.2.1. Dual-Choice Experiment

There were significant effects of treatments in egg counts at χ^2^ (3) = 90.579, *p* < 0.0001 under the dual-choice experiment (Table 1). The post hoc pairwise comparison showed that the tomato plants placed with *R. officinalis* resulted in the lowest egg counts of 39.80 ± 2.21, which was significantly lower than the tomato alone with 73.30 ± 3.22 (z = 8.627, *p* < 0.0001) and tomato plants placed with *A. fruticosus* (50.30 ± 2.54) (z = 3.119, *p* = 0.0098). However, the tomato + *A. schoenoprasum* treatment (45.50 ± 2.39) showed an intermediary effect and did not differ significantly from tomato + *R. officinalis* (z = 1.751, *p* = 0.2974). Likewise, tomato plants placed with *A. fruticosus* and *A. schoenoprasum* had lower egg counts of 50.30 ± 2.54 and 45.50 ± 2.39 compared to tomato alone (73.30 ± 3.22) at z = 5.622, *p* < 0.0001 and z = 6.959, *p* < 0.0001, respectively.

On the other side, significant effects of treatments in leaf damage percentage across the experiment were observed at χ^2^ (3) = 45.625, *p* < 0.0001 (Table 1). The post hoc pairwise comparison revealed that both screened companion plants significantly reduced leaf damage percentage (47.27% ± 4.76% for *A. schoenoprasum* and 33.94% ± 4.54% for *A. fruticosus*) compared to the negative control (66.34% ± 4.70%) at z = 4.603, *p* < 0.0001 and z = 6.418, *p* < 0.0001, respectively. Also, tomato plant placed with *R. officinalis* recorded the lowest mean leaf damage percentage of 21.05% ± 3.82% compared to tomato alone (66.34% ± 4.70%) and tomato + *A. fruticosus* (33.94% ± 4.54%) at z = 6.418, *p* < 0.0001 and z = 4.059, *p* = 0.0003, respectively. However, *A. schoenoprasum* and *A. fruticosus* did not significantly differ in leaf damage percentage, with 47.27% ± 4.76% and 33.94% ± 4.54%, respectively, at z = 2.001, *p* = 0.187. Likewise, there was no significant difference in leaf damage percentages between the positive control (21.05% ± 3.82%) and tomato plants treated with *A. fruticosus* (33.94% ± 4.54%) at z = 2.143, *p* = 0.1396.

#### 3.2.2. Multiple-Choice Experiments

Results showed significant effects of treatments in egg counts at χ^2^ (3) = 44.8134, *p* < 0.0001 under the multiple-choice experiment (Table 2). The post hoc pairwise comparison revealed that the tomato plant placed near to *A. schoenoprasum* and *A. fruticosus* showed low egg counts of 9.98 ± 1.02 and 13.97 ± 1.22, respectively, with no significant difference between them at z = 2.508, *p* = 0.1920. However, despite the fact that the tomato plant placed near *R. officinalis* recorded the lowest egg count (6.89 ± 0.84), there was no significance difference from the tomato plant placed with *A. schoenoprasum* (9.98 ± 1.02) at z = 2.330, *p* = 0.2769. Also, tomato plants placed far from the companion plants (Nc) displayed higher egg counts compared to tomato plants placed near companion plants (Nt) (Table 2). However, tomato plants placed far from companion plants recorded significantly lower egg counts of 31.03 ± 1.88 for *R. officinalis*, 34.63 ± 1.99 for *A. schoenoprasum* and 39.02 ± 2.13 for *A. fruticosus* compared to the negative control (50.48 ± 2.48) at z = 6.169, *p* < 0.0001, z = 4.900, *p* < 0.0001 and z = 3.421, *p* = 0.0144, respectively.

**Figure 6:**
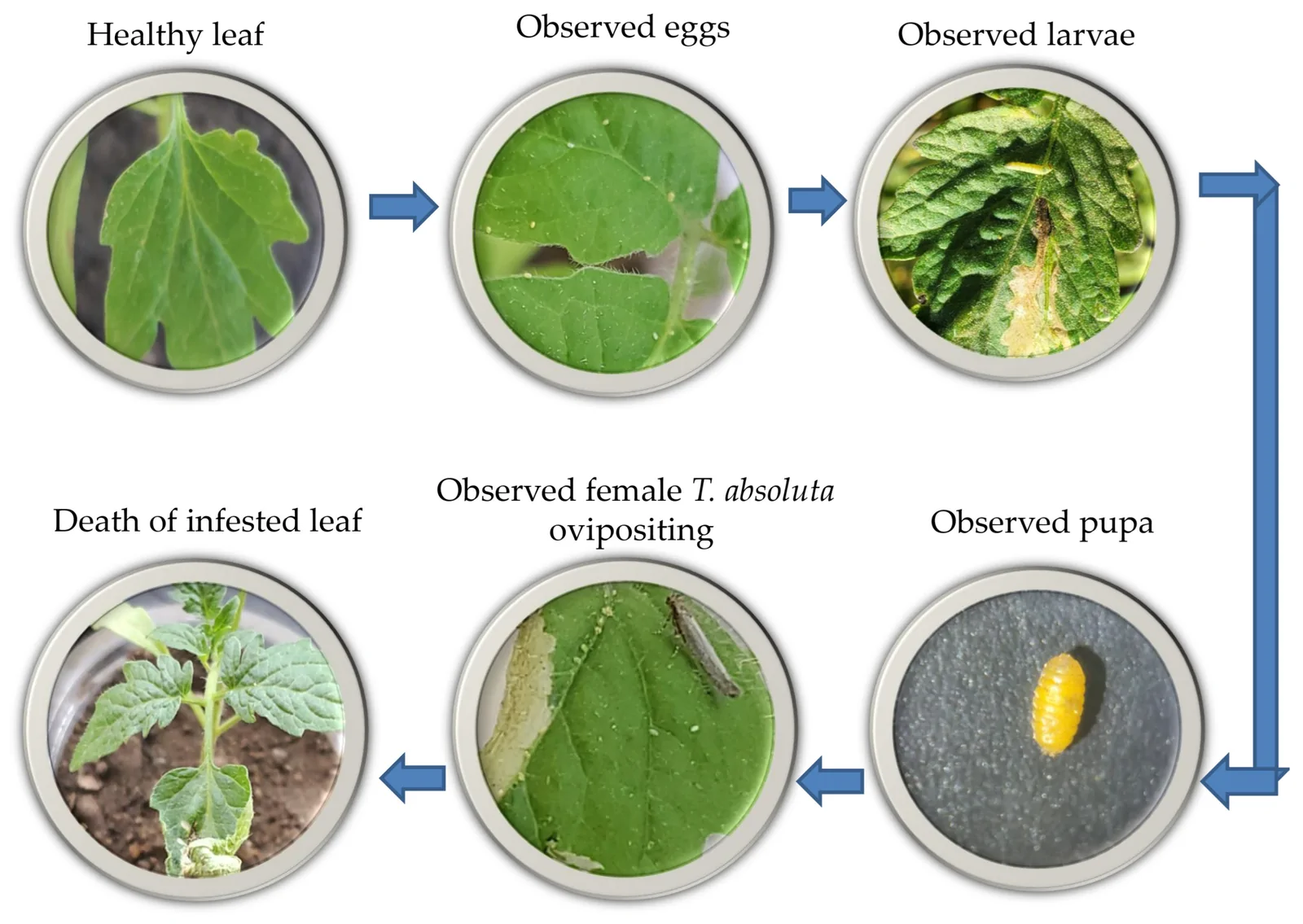
Observed changes in cage experiment consisting of tomato plants and adult female *T. absoluta*.

Likewise, the results indicate significant effects of treatments in leaf damage percentage at χ^2^ (3) = 25.8862, *p* < 0.0001 (Table 2). The post hoc pairwise comparison revealed that all three companion plants placed near to tomato plants showed significantly low leaf damage percentages of 2.47% ± 1.72% for *R. officinalis*, 6.17% ± 2.67% for *A. schoenoprasum* and 11.11% ± 3.70% for *A. fruticosus* compared to tomato alone (51.16% ± 5.39%) at z = 15.082, *p* < 0.0001, z = 14.247, *p* < 0.0001 and z = 12.790, *p* < 0.0001, respectively (Figure 6). Unlike the oviposition results, tomato plants placed near to *A. schoenoprasum* had low leaf damage percentage (6.17% ± 2.67%), with no significant difference from tomato plants placed near *R. officinalis* (2.47% ± 1.72%) at z = 2.330, *p* = 0.2769.

Further analysis indicated that tomato plants placed with *R. officinalis* (positive control) showed the lowest Oviposition Activity Index (−0.64), indicating its high Oviposition Deterrence Index (64%) for the oviposition of female *T. absoluta*. Likewise, tomato plants placed with *A. schoenoprasum* showed intermediary performance over *R. officinalis* and *A. fruticosus*, with 55.26% of ODI and effective repellency of 70.09%. Likewise, the percent effective repellency (77. 57%) recorded in tomato plants placed with *R. officinalis* was significantly higher than tomato placed with *A. fruticosus* and *A. schoenoprasum* (Table 3).

### 3.3. Effects of A. schoenoprasum and A. fruticosus Plant on the Development of T. absoluta in Screenhouse Experiment

The results indicated significant effects of treatments in egg counts at χ^2^ (4) = 189.4, *p* < 0.0001 under the screenhouse experiment (Table 4). The post hoc pairwise comparison revealed that the SNOW TIGER spray (positive control) recorded lower egg counts of 3.71 ± 0.73 compared to the negative control at z = 10.322, *p* < 0.0001. Also, tomato plants placed with *A. schoenoprasum* had lower egg counts of 7.29 ± 1.02, which significantly differed from tomato plants placed with *A. fruticosus* (12.14 ± 1.32) at z = 2.884, *p* = 0.0321. This shows the strong repellency effects of *A. schoenoprasum* under a screenhouse environment. However, both *A. schoenoprasum* and *A. fruticosus* performed significantly better, with lower egg counts compared to the negative control involving tomato alone with water spray (31.57 ± 2.12) at z = 9.439, *p* < 0.0001 and z = 7.487, *p* < 0.0001.

Likewise, a significant effect of treatments in leaf damage percentage was observed at χ^2^ (4) = 51.9156, *p* < 0.0001 (Table 4). The post hoc pairwise comparison revealed that there was no significant difference in leaf damage percentage observed in the positive control (synthetic pesticide) with 9.62% ± 4.09% and tomato plants placed with *A. schoenoprasum* (13.92 ± 3.90) at z = 0.733, *p* = 0.9488. Among the two screened companion plants, *A. schoenoprasum* and *A. fruticosus* did not differ significantly in leaf damage percentages, with 13.92% ± 3.90% and 25.00% ± 4.84%, respectively, at z = 1.742, *p* = 0.4081. However, the integration of *A. fruticosus* and *A. schoenoprasum* did not significantly differ from *A. fruticosus* alone, with leaf damages of 39.44% ± 5.80% and 25.00% ± 4.84%, respectively, at z = 1.889, *p* = 0.3231.

### 3.4. Oviposition Rates Across Treatments

**Figure 7:**
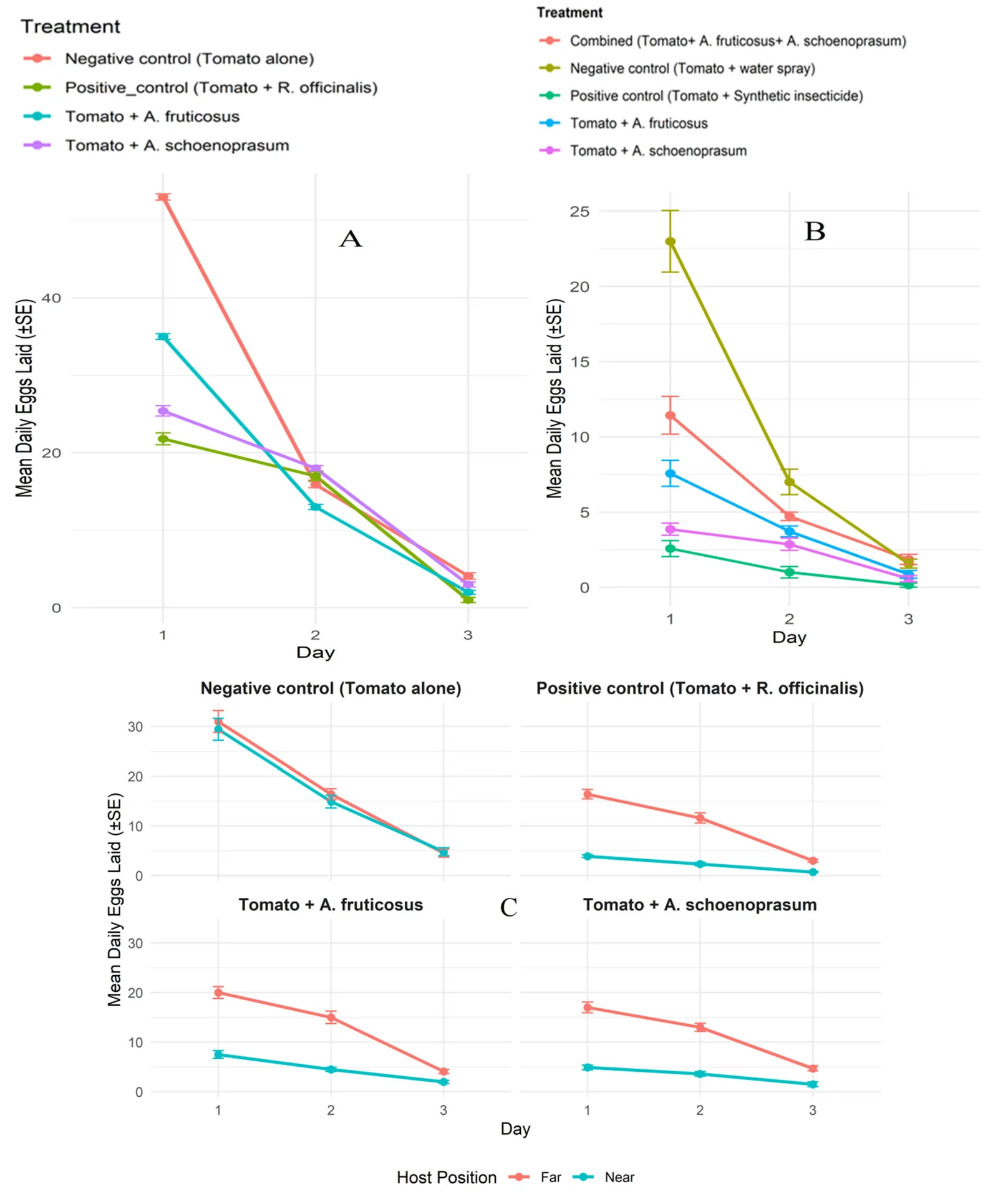
Oviposition rates of adult *T. absoluta* under (**A**) dual-choice, (**B**) screenhouse and (**C**) multiple-choice experiments.

The oviposition rates in all experiments exhibited a downward trend, with peak egg counts on day one followed by dropping on days two and three (Figure 6). However, the normal trend of a sharp drop was observed in negative controls, with 53 eggs laid on day one to 4 eggs on day three for the dual-choice trial (Figure 7A), 30 eggs laid on day one to 5 eggs laid on day three for the multiple-choice trial (Figure 7C) and 23 eggs laid on day one to 2 eggs on day three for the screenhouse trial (Figure 7B).

On the other side, the positive control consisting of tomato plants placed with *R. officinalis* recorded the slowest rate, with a slight drop in eggs laid from day one to day three (22 eggs to 1 egg in dual-choice trial) (Figure 7A) as well as 4 eggs to 1 egg when placed near to *R. officinalis* and 16 eggs to 3 eggs when placed far from *R. officinalis* in the multiple-choice trial (Figure 7C). Likewise, the positive control containing the synthetic insecticide SNOW TIGER in the screenhouse trial recorded the lowest oviposition rate, with a gradual drop in eggs laid, from 3 eggs on day one to 0 eggs on day three compared to a sharp drop observed in the negative control, with 23 eggs on day one to 2 eggs on day three (Figure 7B).

However, in all trials, *A. schoenoprasum* displayed good performances, followed by *A. fruticosus*, in lowering the oviposition rate of *T. absoluta* on tomato plants compared to negative controls. Therefore, *A. schoenoprasum* and *A. fruticosus* showed potential for being incorporated in tomato fields for the management of *T. absoluta*.

### 3.5. Correlation Between Oviposition and Leaf Damage Percentage

**Figure 8:**
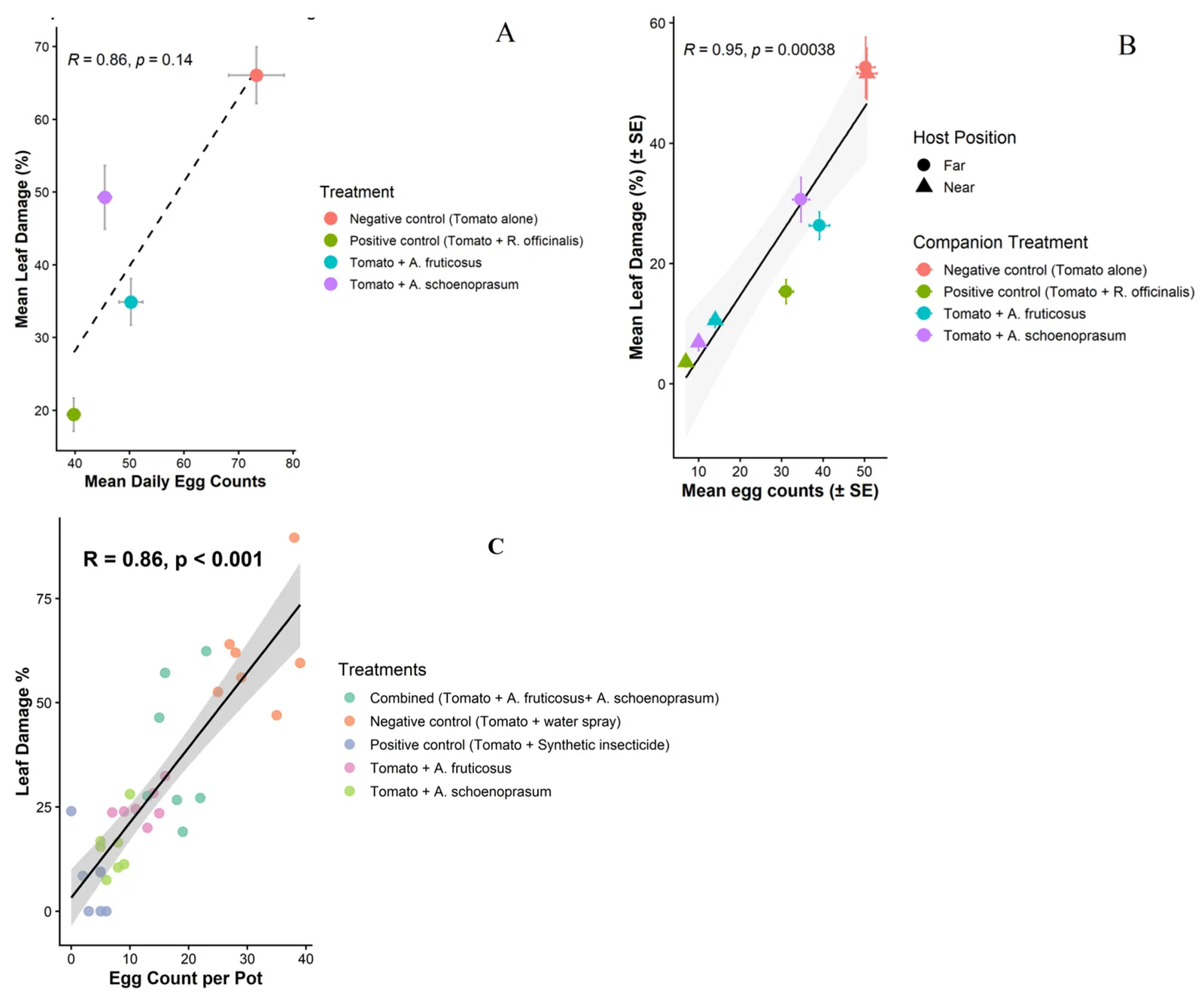
Correlation between mean egg counts and mean leaf damage percentage under (**A**) dual-choice, (**B**) multiple-choice and (**C**) screenhouse experiment.

The results indicated a positive correlation between oviposition and leaf damage percentage in dual-choice, multiple-choice and screenhouse experiments (Figure 8). There was a strong positive correlation between mean egg counts and mean leaf damage percentage at r = 0.86, *p* = 0.14 and *n* = 10 in the dual-choice experiment. However, there was no significant difference in correlation between treatments. Likewise, the multiple-choice trial showed a strong positive correlation at r = 0.95, *p* = 0.00038 and *n* = 10, with a significant difference among treatments. However, the screenhouse experiment exhibited a strong positive correlation at r = 0.86, *p* < 0.001 and *n* = 7 (Figure 8C).

## 4. Discussion

Our study screened fourteen potential companion plants obtained through an extensive literature review and farmer survey study for the ecological management of *T. absoluta* using a Y-tube olfactometer. The two screened companion plants (*A. fruticosus* and *A. schoenoprasum*) were subjected to cage and screenhouse experiments.

The results from the screening experiment indicated that *A. fruticosus* and *A. schoenoprasum* significantly repelled adult *T. absoluta* at *p* < 0.001 (Figure 5). Their performances showed no significant differences from the positive control containing *R. officinalis*. Our findings align with the results reported by Amarawardana et al. [49], who revealed that *A. schoenoprasum* significantly repelled *Myzus persicae* in a Y-tube olfactometer experiment compared to the host plant (*Capsicum annum*). The choices in screening experiment were guided by volatiles emitted from tested plants, indicating the presence of olfactory cues in *A. schoenoprasum* and *A. fruticosus* that subsequently repelled *T. absoluta* adults [61,62].

Also, results from the cage experiment indicated that the tomato plants placed with *A. fruticosus* and *A. schoenoprasum* significantly reduced egg counts compared to tomato alone (Table 1 and Table 2). However, there was no significance difference in egg counts to tomato plants placed with *A. fruticosus* and *A. schoenoprasum*. Similar findings were reported by Åsman et al. [63], who revealed that *A. schoenoprasum* minimized egg counts of *Acrolepiopsis assectella* Zeller on leeks under a cage experiment due to the disruption of host-seeking behaviour. Conversely, results reported by Lai et al. [64] showed that *A. schoenoprasum* attracted *Phytomyza gymnostoma* Loew in cage experiments, indicating the possession of attractive visual and chemical cues. However, the variation in results might be caused by neural-response differences of different insect species to similar olfactory cues [65].

Similarly, our findings showed that the tomato plants intercropped with *A. fruticosus* and *A. schoenoprasum* had lower egg counts and leaf damage percentages compared to the sole tomato plants with no spraying under the screenhouse experiment (Table 4). Compared to the cage experiment, the tomato plants intercropped with *A. schoenoprasum* recorded lower egg counts than tomato plants intercropped with *A. fruticosus*. The differences might be caused by the high content of sulfur compounds emitted from *A. schoenoprasum* [40] that spread to a wider area than the volatiles from *A. fruticosus*. The reduced oviposition in tomato plants intercropped with *A. schoenoprasum* and *A. fruticosus* was possibly caused by the disruption of host-finding mechanisms of *T. absoluta* that subsequently led to reduced leaf damage percentage [66,67,68].

Also, comparable outcomes were noted by Hata et al. [69], showing a reduction in egg counts and leaf damage percentages caused by *Tetranychus urticae* Koch when *A. schoenoprasum* was intercropped with host plants (*Solanum lycopersicum* and *Fragaria ananassa* L.) under a screenhouse environment. The reduction in egg counts on tomato plants intercropped with *A. schoenoprasum* and *A. fruticosus* plays a primary role in lowering the insect pest population [70]. Conversely, the study reported by Held et al. [71] observed no significant effects in the population of *Popillia japonica* Newman on *Rosa × hybrida* hort when integrated with *A. schoenoprasum* and when planted alone (mono-cropping). Similarly, this might be due to variation in the response mechanism of different insect species to similar volatile organic compounds, leading to attractant and repellent effects [65].

Currently, there is no specific study documenting the efficacy of *A. fruticosus* in the management of insect pests under cage, screenhouse and/or field experiments. However, other species of the genus *Aeollanthus* have been studied, with the vast majority of studies relying on laboratory trials focusing on crude essential oils and nano emulsions [72]. The lab-based experiments of the genus *Aeollanthus* limit peer-reviewed articles on its effectiveness in insect pest management when intercropped with other crops. As a member of the Lamiaceae family, *A. fruticosus* has shown potential in repelling *T. absoluta* due to the possession of volatile organic compounds that disrupt the host-finding olfactory mechanism [73].

For example, the volatile profiles of *Aeollanthus pubescens* Benth indicated high composition of fenchone and linacool, which significantly reduced egg counts of *Hypothenemus hampei* Ferrari [74] and lowered the population of public health vectors [75]. Also, recent characterization of the specialized isoppimarane and abietane diterpene space in *Aeollanthus buchnerianus* Briq shows the potential of the genus to produce antifeedant compounds [76].

The succulent nature and adaptability of genus *Aeollanthus* in tropical and subtropical regions give it the potential to be integrated with various crops in diverse agroecological zones [77]. Consequently, future studies should prioritize evaluating the field efficacy of *A. fruticosus*, taking into consideration the volatile organic compound profiles. Therefore, integrating tomato with companion plants plays a key role in promoting ecological balances while suppressing *Tuta absoluta* [78,79]. The limitation of our study is that we did not analyze the volatile organic compounds found in *A. fruticosus*. Therefore, further studies on the analysis and quantification of volatiles from *A. fruticosus* are warranted. Generally, our study demonstrated that *A. fruticosus* and *A. schoenoprasum* are promising repellent plants, indicating their integrated potential and/or ecological management of *Tuta absoluta* in tomato.

## 5. Conclusions

In our study, *A. fruticosus* and *A. schoenoprasum* displayed potential to be used as repellent plants in the management of *T. absoluta*. However, *A. schoenoprasum* displayed the highest performances and was regarded as the most effective companion plant, followed by *A. fruticosus*. Therefore, *A. schoenoprasum* and *A. fruticosus* can be considered potential companion plants for the ecological management of *T. absoluta*.

However, further studies should be conducted to evaluate their effectiveness in the management of *T. absoluta* under field experiments. Also, trials should be performed to assess the integrative performances of *A. fruticosus* and *A. schoenoprasum* with other eco-friendly methods in the management of *T. absoluta*. Likewise, the analysis of chemical compounds from the leaves of *A. fruticosus* should be performed to explore its constituencies and their concentrations.

## Figures and Tables

**Table 1:** Oviposition and leaf damage percentage under dual-choice experiment.

Parameter	Treatment Name	No. of Replicates (N)	Mean ± SE
Oviposition	Positive control (Tomato + *R. officinalis*)	10	39.80 ± 2.21 ^a^
	Tomato + *A. schoenoprasum*	10	45.50 ± 2.39 ^ab^
	Tomato + *A. fruticosus*	10	50.30 ± 2.54 ^b^
	Negative control (Tomato alone)	10	73.30 ± 3.22 ^c^
Leaf damage %	Positive control (Tomato + *R. officinalis*)	10	21.05 ± 3.82 ^a^
	Tomato + *A. schoenoprasum*	10	47.27 ± 4.76 ^b^
	Tomato + *A. fruticosus*	10	33.94 ± 4.54 ^ab^
	Negative control (Tomato alone)	10	66.34 ± 4.70 ^c^

Note: The letter *a*, *b*, *ab* and *c* indicate significant differences (*p* < 0.05, Tukey’s HSD) in mean egg counts and leaf damage percentages.

**Table 2:** Oviposition and leaf damage percentage under multiple-choice experiments.

Parameter	Treatment Name	No. of Replicates (N)	Host Position	Mean ± SE
Oviposition	Positive control (Tomato + *R. officinalis*)	10	Far	31.03 ± 1.88 ^c^
			Near	6.89 ± 0.84 ^a^
	Tomato + *A. schoenoprasum*	10	Far	34.63 ± 1.99 ^c^
			Near	9.98 ±1.02 ^ab^
	Tomato + *A. fruticosus*	10	Far	39.02 ± 2.13 ^c^
			Near	13.97 ± 1.22 ^b^
	Negative control (Tomato alone)	10	Left corner	50.18 ± 2.47 ^d^
			Right corner	50.48 ± 2.48 ^d^
Leaf damage %	Positive control (Tomato + *R. officinalis*)	10	Far	15.56 ± 3.82 ^ab^
			Near	2.47 ± 1.72 ^a^
	Tomato + *A. schoenoprasum*	10	Far	31.25 ± 5.18 ^bc^
			Near	6.17 ± 2.67 ^a^
	Tomato + *A. fruticosus*	10	Far	25.56 ± 4.60 ^b^
			Near	11.11 ± 3.70 ^ab^
	Negative control (Tomato alone)	10	Left corner	51.16 ± 5.39 ^c^
			Right corner	50.00 ± 5.33 ^c^

Note: The letter *a*, *b*, *ab*, *bc*, *c* and *d* indicate significant differences (*p* < 0.05, Tukey’s HSD) in mean egg counts and leaf damage percentages.

**Table 3:** Oviposition parameters under multiple-choice experiments.

Treatments	Treated Tomato (Nt)	Control Tomato (Nc)	Oviposition Activity Index	Oviposition Deterrent Index (%)	Effective Repellency (%)
Tomato + *A. schoenoprasum*	10 ± 3.59 ^a^	34.7 ± 6.38 ^ab^	−0.55 ± 0.14 ^ab^	55.26 ± 14.31 ^ab^	70.09 ± 13.28 ^ab^
Tomato + *R. officinalis*	6.9 ± 1.85 ^a^	31.1 ± 5.45 ^a^	−0.64 ± 0.08 ^a^	63.73 ± 8.36 ^a^	77.57 ± 6.10 ^a^
Tomato + *A. fruticosus*	14 ± 4.24 ^b^	39.1 ± 7.71 ^b^	−0.47 ± 0.16 ^b^	46.98 ± 16.70 ^b^	62.54 ± 16.54 ^b^

Note: The values indicates mean ± standard deviation (SD) (*n* = 10). Mean in the same column followed by different superscript letters are significantly different (*p* < 0.05, Tukey’s HSD).

**Table 4:** Oviposition and leaf damage percentage under screenhouse experiment.

Parameter	Treatment Name	No. of Replicates (N)	Mean ± SE
Oviposition	Positive control (Tomato + Synthetic insecticides)	7	3.71 ± 0.73 ^a^
	Tomato *A. schoenoprasum*	7	7.29 ± 1.02 ^b^
	Tomato + *A. fruticosus*	7	12.14 ± 1.32 ^c^
	Negative control (Tomato alone)	7	31.57 ± 2.12 ^d^
	Combined (Tomato + *A. fruticosus* + *A. schoenoprasum*)	7	18 ± 2.12 ^e^
Leaf damage %	Positive control (Tomato + Synthetic insecticide)	7	9.62 ± 4.09 ^a^
	Tomato *A. schoenoprasum*	7	13.92 ± 3.90 ^a^
	Tomato + *A. fruticosus*	7	25.00 ± 4.84 ^ab^
	Negative control (Tomato alone)	7	63.38 ± 5.72 ^c^
	Combined (Tomato + *A. fruticosus* + *A. schoenoprasum*)	7	39.44 ± 5.80 ^b^

Note: The letter *a*, *b*, *ab*, *c*, *d* and *e* indicate significant differences (*p* < 0.05, Tukey’s HSD) in mean egg counts and leaf damage percentages.

## Data Availability

All data obtained from the study are available in the manuscript and from the authors upon reasonable request.

## Abbreviations

The following abbreviations are used in this manuscript:

VOC	Volatile organic compound
MUST	Mbeya University of Science and Technology
ODI	Oviposition Deterrent Index
RH	Relative humidity
